# Risk for Death among Children with Pneumonia, Afghanistan

**DOI:** 10.3201/eid2308.151550

**Published:** 2017-08

**Authors:** Rahmani Zabihullah, Bhim G. Dhoubhadel, Ferogh A. Rauf, Sahab A. Shafiq, Motoi Suzuki, Kiwao Watanabe, Lay M. Yoshida, Michio Yasunami, Salihi Zabihullah, Christopher M. Parry, Rabi Mirwais, Koya Ariyoshi

**Affiliations:** Nagasaki University, Nagasaki, Japan (R. Zabihullah, B.G. Dhoubhadel, M. Suzuki, K. Watanabe, L.M. Yoshida, M. Yasunami, C.M. Parry, K. Ariyoshi);; Abu Ali Sina Balkhi Regional Hospital, Mazar-e-Sharif, Afghanistan (F.A. Rauf, S.A. Shafiq, S. Zabihullah);; London School of Hygiene and Tropical Medicine, London, UK (C.M. Parry);; Public Health Department, Balkh Province, Afghanistan (R. Mirwais)

**Keywords:** Afghanistan, anemia, child, malnutrition, mortality, pneumococcal infections, pneumococcal vaccines, pneumonia, serogroup, Streptococcus pneumoniae, bacteria, Japan, United Kingdom

## Abstract

In Afghanistan, childhood deaths from pneumonia are high. Among 639 children at 1 hospital, the case-fatality rate was 12.1%, and 46.8% of pneumococcal serotypes detected were covered by the 13-valent vaccine. Most deaths occurred within 2 days of hospitalization; newborns and malnourished children were at risk. Vaccination could reduce pneumonia and deaths.

In Afghanistan, the mortality ratio for children <5 years of age is 90 deaths/1,000 live births, twice the global average; 20% of deaths are from pneumonia (*1*). Although Afghanistan is considered 1 of the 5 countries with the highest level of childhood deaths from pneumonia, studies of the risk factors for death and etiology of pneumonia among children in Afghanistan are lacking (*2*). We therefore determined risk factors for death from pneumonia in children <5 years of age in a regional hospital in Afghanistan and the distribution of pneumococcal serotypes carried in the nasopharynx.

## The Study

From December 2012 through the second week of March 2013, we conducted a prospective observational study in the Department of Pediatrics in Abu Ali Sina Balkhi Regional Hospital, Mazar-e-Sharif, Afghanistan, a 700-bed regional referral hospital for Balkh Province. This study was conducted before pneumococcal conjugate vaccine 13 (PCV13) had been introduced in Afghanistan. We enrolled children <5 years of age who met the World Health Organization (WHO) criteria for clinical pneumonia at the time of admission (*3*). We collected data by standardized questionnaire and determined immunization status by history, immunization report, and bacillus Calmette–Guérin (BCG) scar.

Malnutrition was defined as weight-for-age *z* score <−2 (WHO Anthro software version 3.2.2, http://www.who.int/childgrowth/software/en/). Anemia in children >6 months of age was determined by hemoglobin cutoff values established by WHO (*4*) and in children <6 months by hemoglobin value <2 SDs below the mean for age group (*5*). Illness severity was classified by WHO criteria (*3*). Hospital outcomes were classified as discharged (discharged after successful treatment), deceased (died during hospitalization), and unknown (still in hospital at study end). Children with unknown status were excluded from risk factor analysis. Neonates/newborns and infants were defined as children <1 and <12 months of age, respectively.

We collected nasopharyngeal samples according to the WHO protocol by using flocked swabs (Copan Diagnostics, Murireta, CA, USA) that were stored in STGG (skim milk, tryptone, glucose, and glycerine) media at −10°C and transferred within 1 month by airplane with cold ice packs in a thermos to Nagasaki, Japan, where they were stored at −20°C. DNA was extracted by using a QIAamp DNA Blood Mini Kit (QIAGEN, Hilden, Germany). *Streptococcus pneumoniae* was detected by lytA real-time PCR (selective for autolysin gene), and serotyping was performed by a nanofluidic real-time PCR that detects 50 serotypes as individual serotype/serogroup including all vaccine serotypes (*6*). 

We considered samples positive by lytA PCR but negative for serotypes/serogroups nontypeable and co-colonization with multiple serotypes to be presence of >2 serotypes/serogroups in a sample. The percentage of vaccine serotypes was calculated as the proportion of samples that had a vaccine serotype (including minor serotypes in co-colonization) among samples positive by lytA PCR.

For analyses we used Epi Info version 7 (Centers for Disease Control and Prevention, Atlanta, GA, USA) and Stata 12 (StataCorp LLP, College Station, TX, USA). In the univariate analysis model, we included risk factors for death from pneumonia at p<0.2, and in the multivariate model we included age, sex, and ethnicity. The study complied with STROBE (https://strobe-statement.org/fileadmin/Strobe/uploads/checklists/STROBE_checklist_v4_combined.pdf) guidelines and was approved by the Nagasaki University Institutional Review Board, Nagasaki, Japan, and the Balkh Public Health Department, Balkh, Afghanistan. Written informed consent was obtained from the parents of enrolled children.

**Figure 1:**
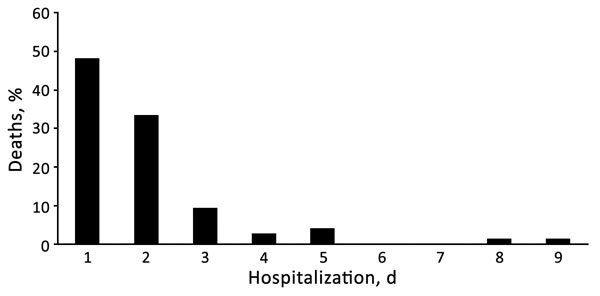
Proportion of deaths and days of hospitalization among children <5 years of age with pneumonia admitted to Abu Ali Sina Balkhi Regional Hospital, Mazar-e-Sharif, Afghanistan, December 2012–March 2013.

Parents of 670 children were approached, and 639 children were enrolled (Technical Appendix Figure 1). Median patient age was 5.0 (interquartile range 2.5–9.0) months; 82.5% were infants and 64.3% were male (Table 1). Pneumonia case-fatality ratio (CFR) was 12.1% (75/617; 95% CI 9.6%–14.9%) (Technical Appendix Table 1). A total of 61 (81.3%) children died within 2 days of hospitalization (Figure 1), and most were infants (Technical Appendix Figure 2).

**Table 1:** Characteristics and clinical outcomes for 639 children <5 years of age with pneumonia hospitalized at Ali Sina Balkhi Regional Hospital, Mazar-e-Sharif, Afghanistan, December 2012–March 2013

Characteristics	No. (%)
Sex	
M	411 (64.3)
F	228 (35.7)
Age, mo	
<1 (newborn)	17 (2.7)
1–11	510 (79.8)
>12	112 (17.5)
Maternal illiteracy	549 (85.9)
Duration of illness >7 d before hospitalization	102 (16.0)
Ethnicity	
Tajik	300 (46.9)
Pashtoon	123 (19.3)
Uzbek	77 (12.0)
Hazara	101 (15.8)
Other	38 (6.0)
Received antimicrobial drugs before hospitalization	
Yes	561 (87.8)
No	65 (10.2)
Unknown	13 (2.0)
Vaccination status, vitamin A intake, and nutritional status	
Bacillus Calmette–Guérin vaccine	545 (85.2)
>1 dose of pentavalent vaccine, n = 568	436 (76.8)
Measles vaccine, n = 171	107 (62.6)
>1 dose of vitamin A, n = 345	119 (34.5)
Malnutrition*	
Detected	255 (39.9)
Not detected	381 (59.6)
Not evaluated	3 (0.5)
Anemia†	
Detected	296 (46.3)
Not detected	220 (34.4)
Not evaluated	123 (19.3)
Both malnutrition and anemia, n = 514	126 (24.5)
Very severe pneumonia‡	532 (83.3)
Clinical outcome, n = 617	
Discharged well	542 (87.9)
Death	75 (12.1)

*Defined by weight/age *z* score <–2, and the *z* score value was calculated by using World Health Organization Anthro software, version 3.2.2 9 (http://www.who.int/childgrowth/software/en/). †Defined according to the World Health Organization (*4*) and Janus et al. (*5*). ‡Severity classified according to World Health Organization (*3*). Very severe pneumonia is defined as cough or difficulty breathing with 1 of the following: central cyanosis, difficulty feeding, convulsions, lethargy, loss of consciousness, severe respiratory distress.

According to univariate analysis, risk for death was increased among newborn (odds ratio [OR] 11.1) and malnourished (OR 2.06) children (Table 2). Protective factors were receipt of BCG vaccine (OR 0.39), >1 dose of pentavalent vaccine (OR 0.53), and vitamin A (OR 0.39). Among malnourished children, female sex was associated with death (Technical Appendix Table 2). We found no significant differences by sex in terms of epidemiologic, clinical, and nutritional status (data not shown). BCG vaccination was independently associated with decreased risk for death among patients with pneumonia (Table 2) and among malnourished children with pneumonia (Technical Appendix Table 2).

**Table 2:** Risk factors for death from pneumonia among children <5 years of age hospitalized at Abu Ali Sina Balkhi Regional Hospital, Mazar-e-Sharif, Afghanistan, December 2012–March 2013

Variable	Discharged, no. (%), n = 542	Deceased, no. (%), n = 75	Odds ratio (95% CI)	p value	Adjusted odds ratio (95% CI)*	p value
Sex						
F	186 (34.3)	32 (42.7)	1.42 (0.87–2.32)	0.15	1.61 (0.96–2.71)	0.06
M	356 (65.7)	43 (57.3)	1		1	
Age, mo						
<1 (newborn)	9 (1.7)	8 (10.7)	11.1 (3.36–36.6)	<0.01	13.1 (3.71–46.5)	<0.01
1–11	433 (89.9)	59 (78.6)	1.70 (0.78–3.67)	0.17	1.59 (0.72–3.49)	0.24
>12	100 (18.4)	8 (10.7)	1			
Maternal illiteracy						
Literate	83 (15.3)	7 (9.3)	0.56 (0.25–1.28)	0.17	0.62 (0.26–1.45)	0.27
Illiterate	459 (84.7)	68 (90.7)	1			
Duration of illness, d						
>7	80 (14.8)	17 (22.7)	1.56 (0.85–2.85)	0.14	1.73 (0.92–3.25)	0.08
<7	462 (85.2)	58 (77.3)	1			
Ethnicity						
Tajik	252 (46.5)	35 (46.7)	1			
Pashtoon	105 (19.4)	16 (21.3)	1.09 (0.58–2.06)	0.77	0.80 (0.40–1.60)	0.54
Uzbek	62 (11.5)	10 (13.3)	1.16 (0.54–2.47)	0.69	1.04 (0.47–2.31)	0.91
Hazara	89 (16.4)	11 (14.7)	0.88 (0.43–1.82)	0.75	0.81 (0.38–1.72)	0.58
Other	34 (6.3)	3 (4.0)	0.63 (0.18–2.17)	0.47	0.48 (0.13–1.75)	0.27
Received antimicrobial drugs before hospitalization						
Yes	476 (87.8)	65 (86.7)	1.09 (0.47–2.49)	0.83		
No	56 (10.3)	7 (9.3)	1			
Unknown	10 (1.9)	3 (4.0)	2.4 (0.52–10.9)	0.24		
Bacillus Calmette–Guérin vaccine						
Received	474 (87.4)	55 (73.3)	0.39 (0.22–0.69)	<0.01	0.47 (0.25–0.88)	0.02
Not received	68 (12.6)	20 (26.7)	1			
>1 dose pentavalent vaccine, n = 547†						
Received	380 (79.0)	44 (66.7)	0.53 (0.30–0.92)	0.02		
Not received	101 (21.0)	22 (33.3)	1			
Measles vaccine, n = 165†						
Received	98 (64.9)	6 (42.9)	0.40 (0.13–1.23)	0.11		
Not received	53 (35.1)	8 (57.1)	1			
Vitamin A, n = 336†						
Received	111 (37.1)	7 (18.9)	0.39 (0.16–0.92)	0.03		
Not received	188 (62.9)	30 (81.1)	1			
Malnutrition‡						
Detected	199 (36.7)	41 (54.7)	2.06 (1.26–3.35)	<0.01	2.06 (1.22–3.49)	<0.01
Not detected	340 (62.7)	34 (45.3)	1			
Anemia						
Detected	246 (45.4)	39 (52.0)	1.32 (0.76–2.29)	0.31		
Not detected	192 (35.4)	23 (30.7)	1			
Not evaluated	104 (19.2)	13 (17.3)	1.04 (0.50–2.14)	0.90		

*Age, sex, ethnicities and variables with p<0.2 in univariate analyses were included in multivariate analysis; blank cells indicate variables without p<0.2 in univariate analysis. Because they had collinearity, bacillus Calmette–Guérin vaccine was included with vaccines and vitamin A was included in multivariate analysis.  †No. children eligible for the vaccines or vitamin A. ‡3 children, whose nutritional status was unknown, were excluded.

**Figure 2:**
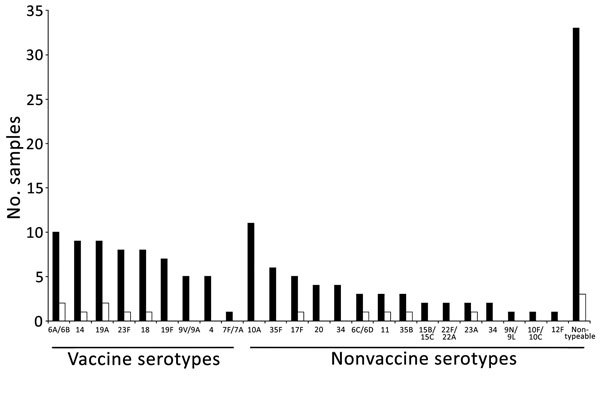
Number of nasopharyngeal samples and pneumococcal serotype/serogroup distribution (including minor serotypes in multiple serotypes) among 110 discharged (black bars) and 11 deceased (white bars) children with pneumonia admitted to Abu Ali Sina Balkhi Regional Hospital, Mazar-e-Sharif, Afghanistan, December 2012–March 2013.

We obtained nasopharyngeal samples from 326 children (Technical Appendix Figure 1). From half (49.9%) of the children, samples could not be taken because of disease severity; CFR was 18.3% for these children, who were more likely to be malnourished and to have received antimicrobial drugs before hospitalization (Technical Appendix Table 3). *S. pneumoniae* was detected in 124 (38.0%) of the 326 samples; 24 serotypes/serogroups were identified (Figure 2). Most (87.8%) children had received antimicrobial drugs before admission, which, along with difficulty storing samples at the research site, influenced detection of pneumococci. The proportions of colonization were 35.9% among children who received antimicrobial drugs before hospitalization and 48.8% among children who did not (p = 0.11). The proportion of samples that had serotypes covered by 7-, 10-, and 13-valent pneumococcal conjugate vaccines were 39.5%, 39.5% and 46.8%, respectively. Co-colonization with multiple serotypes occurred in 21 (16.9%) of 124 positive samples.

## Conclusions

The CFR for children <5 years of age with pneumonia admitted to a regional hospital in Afghanistan was 12.1%, compared with only 7.6% for the full WHO Eastern Mediterranean region (*7*). Most deaths occurred within 2 days of hospitalization. Factors that may have contributed to the high mortality rate were delays in presentation to healthcare facilities, inability to identify severe symptoms in children, and delayed referral from primary care (*7*). These issues could be addressed by strengthening the Integrated Management of Childhood Illness program of WHO, introduced in Afghanistan in 2004 (*8*).

Our finding that newborns and children with malnutrition were at increased risk for death is consistent with findings of studies in India and Pakistan (*9*,*10*). These risks could be reduced by use of clean delivery kits, clean delivery practices, exclusively breast-feeding, education about complementary feeding, and provision of complementary foods in regions where food is less secure (*11*,*12*). 

BCG vaccination was protective. Neonatal BCG vaccination is known to be associated with reduced rates of childhood death, respiratory infection, and sepsis, probably by nonspecific immune effects (*13*); it can also be a proxy for better access to healthcare, immunization, and unmeasured favorable factors. 

Female sex was significantly associated with death among malnourished children, which was not explained by association with other variables. A higher incidence of acute lower respiratory infection in male children, particularly in southern Asia, was reported in a recent systematic review, but the pneumonia CFR was higher in girls than in boys <1 year of age (*7*).

With support from the Global Vaccine Alliance, WHO, and the United Nations Children’s Fund, PCV13 was introduced in Afghanistan in January 2014. Our study detected a wide variety of serotypes, including nonvaccine serotypes. Vaccine coverage was comparable with that found by regional studies (*14*,*15*). Serotype data are limited by the short study duration, common use of antimicrobial drugs before hospitalization, and difficult storage of samples.

The high rate of death from pneumonia among children could be reduced by strengthening existing public health programs (e.g., Integrated Management of Childhood Illness, nutrition programs, and immunization programs). Although the proportion of serotypes covered by PCV13 vaccines was 46.8%, PCV13 could still prevent many cases of pneumonia and deaths among children in Afghanistan.

Technical Appendix Case-fatality ratios, risk factors for death, characteristics, and outcomes for children <5 years of age admitted to Abu Ali Sina Balkhi Regional Hospital with pneumonia, Mazar-e-Sharif, Afghanistan, December 2012–March 2013.
