# Supplementary material for: Risk for Death among Children with Pneumonia, Afghanistan
Source: Emerg Infect Dis. 2017 Aug;23(8):1404–8. doi: 10.3201/eid2308.151550 (PMC5547795; doi:10.3201/eid2308.151550)
Supplement: Technical Appendix — Case-fatality ratios, risk factors for death, characteristics, and outcomes for children <5 years of age admitted to Abu Ali Sina Balkhi Regional Hospital with pneumonia, Mazar-e-Sharif, Afghanistan, December 2012–March 2013. [file 15-1550-Techapp-s1.pdf]

Article DOI: https://doi.org/10.3201/eid2308.151550

Risk for Death among Children with Pneumonia, Afghanistan

Technical Appendix

Technical Appendix Table 1. Case fatality ratio (CFR) stratified among relevant demographic and clinical characteristics

Characteristics	Case Fatality Ratio (CFR) [X100%]
Sex	
Male	0.10
Female	0.14
Age	
<1 mo (newborn)	0.47
1–11 mo	0.12
≥12 mo	0.07
Maternal education	
Literate	0.07
Illiterate	0.12
Duration of illness >7 d before hospitalization	
≤ 7 d	0.11
>7 d	0.16
Ethnicity	
Tajik	0.12
Pashtoon	0.13
Uzbek	0.13
Hazara	0.11
Received antimicrobial drugs before hospitalization	
Yes	0.11
No	0.12
Vaccination status, vitamin A intake and nutritional status	
Bacillus Calmette–Guérin	
Yes	0.10
No	0.22
At least 1 dose of pentavalent (DTwP+Hib+HepB)	
Received	0.10
Not received	0.17
Measles	
Received	0.05
Not received	0.13
At least 1 dose of vitamin A	
Received	0.05
Not received	0.13
Malnutrition	
Detected	0.17
Not detected	0.09
Anemia	
Detected	0.13
Not detected	0.10
Both malnutrition and anemia (N = 514)	
Detected	0.21
Not detected	0.10
Very severe pneumonia[‡]	
Yes	0.14
No	0.00

Technical Appendix Table 2. Risk factors for death due to pneumonia among malnourished under-five children who were admitted pneumonia cases

Variable	Discharged N = 199 (%)	Deceased N = 41 (%)	Odds ratio (OR) (95% CI)	p value	Adjusted OR (95% CI)	p value
Sex						
Female	69 (34.7)	20 (48.8)	1.79 (0.91–3.53)	0.09	2.15 (1.04–4.42)	0.03
Male	130 (65.3)	21 (51.2)	1		1	
Age						
<1 mo (newborn)	1 (0.5)	2 (4.9)	15.0 (1.09–205)	0.04	11.0 (0.64–189)	0.09
1–11 mo	168 (84.4)	35 (85.4)	2.56 (0.51–4.71)	0.42	1.51 (0.48–4.70)	0.45
≥ 12 mo	30 (15.1)	4 (9.7)	1			
Maternal illiteracy						
Literate	31 (15.6)	3 (7.3)	0.42 (0.12–1.47)	0.17	0.44 (0.12–1.62)	0.22
Illiterate	168 (84.4)	38 (92.7)	1			
Duration of illness						
>7 d	31 (15.6)	9 (21.9)	1.52 (0.66–3.50)	0.32		
≤ 7 d	168 (84.4)	32 (78.1)	1			
Ethnicity						
Tajik	82 (41.2)	16 (39.0)	1			
Pashtoon	45 (22.6)	12 (29.3)	1.36 (0.59–3.14)	0.46	0.93 (0.37–2.29)	0.87
Uzbek	25 (12.6)	6 (14.6)	1.23 (0.43–3.47)	0.69	1.15 (0.38–3.40)	0.79
Hazara	34 (17.1)	6 (14.6)	0.90 (0.32–2.50)	0.84	0.72 (0.24–2.13)	0.56
Other	13 (6.5)	1 (2.5)	0.39(0.04–3.23)	0.38	0.20 (0.02–1.81)	0.15
Received antimicrobial drugs before hospitalization						
Yes	176 (88.4)	39 (95.1)	2.10 (0.47–9.41)	0.33		
No	19 (9.6)	2 (4.9)	1			
Unknown	4 (2.0)	0 (0.0)				
BCG vaccine						
Received	161 (80.9)	26 (63.4)	0.40 (0.19–0.84)	<0.01	0.37 (0.17–0.82)	<0.01
Not received	38 (19.1)	15 (36.6)	1			
Penta-valent vaccine at least a dose (N = 218)*						
Received	126 (70.4)	22 (56.4)	0.54 (0.26–1.10)	0.09		
Not received	53 (29.6)	17 (43.6)	1			
Measles vaccine (N = 57) *						
Received	23 (45.1)	2 (33.3)	0.60 (0.10–3.62)	0.58		
Not received	28 (54.9)	4 (66.7)	1			
Vitamin A (N = 118)*						
Received	36 (37.9)	2 (8.7)	0.15 (0.34–0.70)	<0.01		
Not received	59 (62.1)	21 (91.3)	1			
Anemia						
Detected	93 (46.7)	25 (61.0)	1.78 (0.82–3.86)			
Not detected	73 (36.7)	11 (26.8)	1			
Not evaluated	33 (16.6)	5 (12.2)	1.00 (0.32–3.12)			

*Number of eligible children for the vaccines or vitamin A. Penta-valent vaccine, measles vaccine and vitamin A intake were not included in the final model; they have co-linearity with BCG vaccine.

Technical Appendix Table 3. Characteristics of children with the nasopharyngeal samples taken and not taken.

Characteristics	Nasopharyngeal samples taken		p value
	No, N = 313 (%)	Yes, N = 326 (%)	
Male sex	198 (63.3)	213 (65.3)	0.58
Age			
<1 mo (newborn)	11 (3.5)	6 (1.8)	0.17
1–11 mo	254 (81.2)	256 (78.6)	
≥ 12 mo	48 (15.3)	64 (19.6)	
Maternal illiteracy	276 (88.2)	273 (83.7)	0.10
Duration of illness >7 d before hospitalization	54 (17.2)	48 (14.7)	0.38
Ethnicity			
Tajik	135 (43.1)	165 (50.6)	0.20
Pashtoon	65 (20.8)	58 (17.8)	
Uzbek	44 (14.1)	33 (10.1)	
Hazara	53 (16.9)	48 (14.7)	
Others	16 (5.1)	22 (6.8)	
Received antimicrobial drugs before hospitalization			
Yes	285 (91.0)	276 (84.7)	0.04

Characteristics	Nasopharyngeal samples taken No, N = 313 (%)	Nasopharyngeal samples taken Yes, N = 326 (%)	p value
No	24 (7.7)	41 (12.6)	
Unknown	4 (1.3)	9 (1.7)	
Vaccination status and vitamin A intake			
BCG	262 (83.7)	283 (86.8)	0.26
At least a dose of penta-valent (DTwP+Hib+HepB) (n = 568)*	201 (74.7)	235 (78.6)	0.27
Measles (n = 171)*	39 (54.2)	68 (68.7)	0.05
At least a dose of vitamin A received (n = 345)*	50 (33.8)	69 (35.0)	0.81
Clinical features			
Malnutrition (Weight/Age, *z* <-2)			
Detected	143 (45.7)	112 (34.4)	<0.01
Not detected	168 (53.7)	213 (65.3)	
Not evaluated	2 (0.6)	1 (0.3)	
Anemia			
Detected	139 (44.4)	157 (48.2)	0.63
Not detected	112 (35.8)	108 (33.1)	
Not evaluated	62 (19.8)	61 (18.7)	

*Number of eligible children for the vaccines or vitamin A.

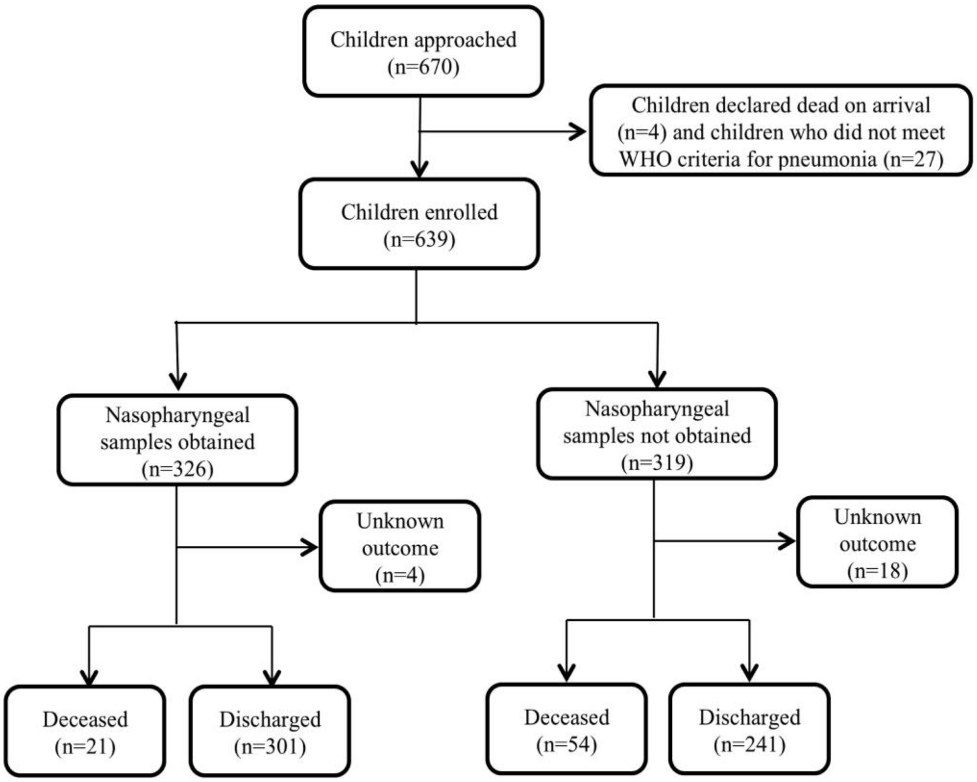

Technical Appendix Figure 1. Profile of children enrolled in the study with their nasopharyngeal samples.

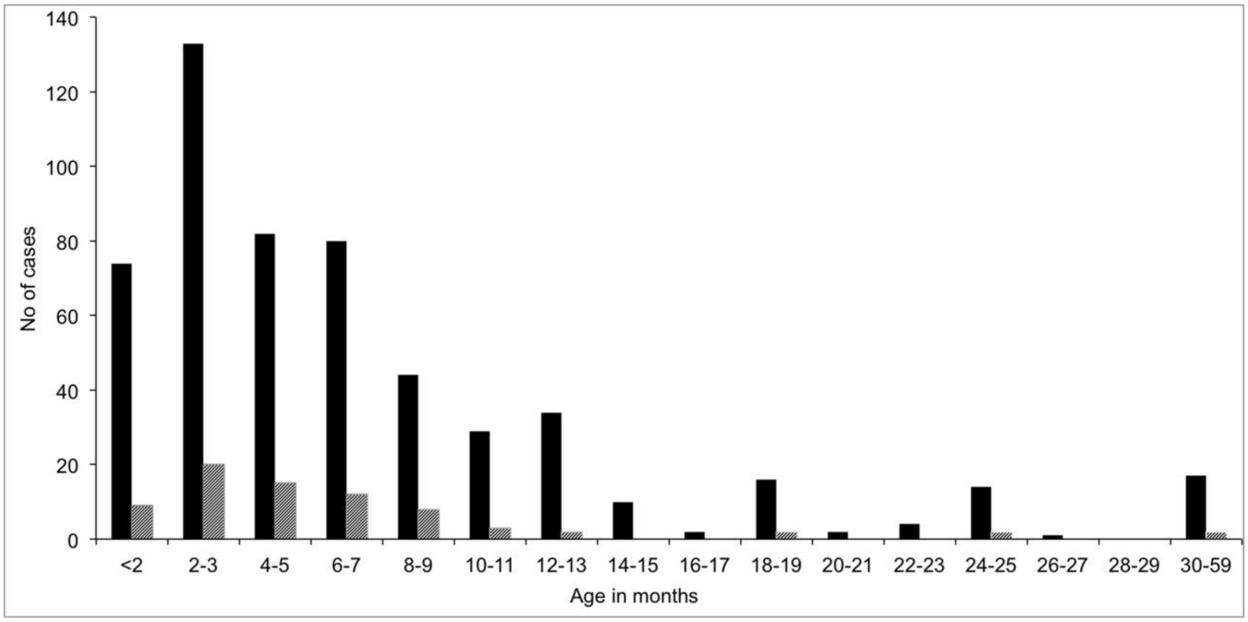

Technical Appendix Figure 2. Distribution of discharged and death cases in different age groups. Age group <2 months includes newborns (<1 month).
